# Peculiarities of occupational mental health care in kindergarten teachers

**DOI:** 10.3389/fpsyg.2023.1168300

**Published:** 2023-06-29

**Authors:** Zhansaya Otarbayeva, Bibianar Baizhumanova, Ulbossyn Tuyakova, Aliya Mambetalina, Assem Umirzakova, Lyazzat Kulzhabayeva

**Affiliations:** ^1^Department of Psychology, L.N. Gumilyov Eurasian National University, Nur-Sultan, Kazakhstan; ^2^Department of Pedagogy, Psychology and Primary Education, Aktobe Regional University K. Zhubanova, Aktobe, Kazakhstan

**Keywords:** depression, mental health recommendations, pedagogical competence, well-being, workplace stress

## Abstract

The purpose of this paper is to quantify the factors that disrupt the mental health of kindergarten (KG) teachers. For this, the researchers conducted an electronic survey of preschool teachers (*n* = 587) on a popular educational platform with the Symptom Checklist-90-R and content analysis of interviews in practicing KG teachers (*n* = 105) with an open discussion of the main stressors during professional activities. Self-reports indicated that depression, interpersonal sensitivity, and anxiety were the main mental health symptoms. ANOVA has revealed that total teaching experience is a statistically significant factor for the mental health of KG teachers: *F*(2.60) = 5.99. According to respondents, the main stressors included concern for the children’s health, fear of injuries, and difficulties in communicating with parents. The synthesis of results allowed for proposing six specific steps for mental health care in KG teachers. The findings are important for administrators and officials of preschool education. The proposed approach can become a theoretical basis for finding ways of mental health care for practicing teachers in further research.

## 1. Introduction

The teacher’s occupational well-being is a positive state of health when his/her expectations are balanced by the needs of students: the teacher is satisfied with his/her work and systematically achieves his/her goals by collaborating with students and colleagues (Na’imah et al., 2021). Mental health is an important component of psychological well-being. Based on the definition by the World Health Organization, mental health is a state of well-being that allows a person to successfully cope with daily stresses and work productively (Huang et al., 2020).

Occupational cognitive and emotional load as a result of working with young children leads to mental health disorders in KG teachers (Koulierakis et al., 2019) resulting in depression, anxiety, and somatization. KG teachers are reported to have the highest depression compared to other occupational categories (Faulkner et al., 2016); they are constantly emotionally stressed and suffer from burnout (Čecho et al., 2019). The mental health of KG teachers determines their performance (Huang et al., 2020). Despite this, detailed information on the mental health of KG teachers with practical recommendations for its preservation is very limited, the problems underlying occupational stress are poorly understood, and the existing literature is disproportionately focused on Western developed societies. At the same time in less developed Asian and African countries, the prerequisites and consequences of KG teachers’ mental health are poorly documented (Huang et al., 2020). This article aims to fill this gap and describes stresses that can affect the mental health of kindergarten teachers during their professional activities.

The predictors of KG teachers’ mental well-being can be divided into personal, professional, and organizational (Na’imah et al., 2021). The most important personal factors are demographic variables such as marital status and age (Koulierakis et al., 2019), self-efficacy, and self-confidence (Bermejo-Toro et al., 2016); the most relevant professional factors are experience, knowledge, skills, and workload (Na’imah et al., 2021); the most significant organizational factors are the work environment, salary level (Koulierakis et al., 2019), organizational structure, and team climate (Parent-Lamarche and Marchand, 2019). Low salaries, lack or insufficiency of benefits and social guarantees, difficult working conditions, and expectations that do not correspond to the real situation are some of the frequently reported stress moderators for KG teachers (Faulkner et al., 2016). Educators often lack adaptive or maladaptive skills to deal with stress. While clearly stating a problem and seeking support in solving it can mitigate stress, teachers often resort to inadequate ways of dealing with occupational stress (avoidance, ignoring, and emotion-focused coping) due to the absence of developed mechanisms for coping with stressful situations (Čecho et al., 2019). It is worth noting that, despite numerous predictors for psychological problems, KG teachers often get pleasure from communicating with children and job satisfaction (Faulkner et al., 2016; Magen-Nagar and Firstater, 2019; Gu et al., 2020).

Faulkner et al. (2016) described a content analysis of interviews with 7 focus groups of KG teachers in Central Texas (United States) to find out their main sources of occupational stress and the impact of these sources on their mental well-being. Faulkner et al. (2016) provide important information but having only open-ended interview questions can be a gap as not all KG teachers can formulate their responses as objectively as possible.

According to Koulierakis et al. (2019), the burnout levels of KG teachers were recorded and the parameters of their quality of life were assessed; a high correlation was found between professional burnout and the quality of life. The quality of life (Koulierakis et al., 2019) was evaluated with the following subscales: Social relationships, Physical health, Psychological health, and Environment.

Čecho et al. (2019) studied the stress level of KG teachers and indicated the psychosocial risks associated with professional activities. Čecho et al. (2019) found that KG teachers, who are not burdened by their professional activities, are more satisfied with their professional results and do not suffer from lack of time. Fináncz et al. (2020) synthesized previous studies and in addition to the Beck Depression Questionnaire and Maslach Burnout Questionnaire proposed their questions to explore the characteristics of occupational well-being and mental health of early childhood educators. Fináncz et al. (2020) found a close connection between depression and work experience in the field. In addition, Fináncz et al. (2020) reported significant differences between specialists working in nurseries and kindergartens: the atmosphere of the nursery turned out to be less favorable for the mental health of educators (Fináncz et al., 2020). Li and Li (2020) studied the effect of self-control and social support on occupational stress in KG teachers and showed a strong relationship between occupational stress and self-control, especially for those respondents, who complained about low social support.

A literature review showed that mental health care of KG teachers begins with the identification and analysis of the factors that determine their psychological problems as a result of professional activities. After a constructive synthesis of factors, professional development programs for KG teachers can be created and implemented with an emphasis on supporting mental health. Thus, this study aims to quantify the factors that impair the mental health of KG teachers in the workplace in developing countries and outline ways to preserve it.

## 2. Materials and methods

### 2.1. Research design

The first research stage is to identify the main mental pathologies associated with professional activities and to determine differences depending on teaching experience (less than a year, 1–3 years, 3–5 years, 5–10 years, and more than 10 years), educational level (secondary/higher), income (low/medium/high), and kindergarten attribute (public/private). The second stage is to reveal the main stressors in the workplace. The third stage is to formulate recommendations for maintaining mental health in the professional activities of KG teachers based on the synthesis of the current findings and previous studies from international practice.

The researchers adopted a scheme, according to which the psychological problems of KG teachers result from personal and organizational factors. They measured such factors by a questionnaire on mental disorders and occupational stresses directly associated with workplace activities and performed an interview content analysis.

### 2.2. Participants

The first stage enrolled users of an English-language educational platform for preschool teachers and administrators, who expressed their desire to participate and received a link to the questionnaire. The only selection criterion was a current work as a KG teacher in developing countries of Europe/Asia/Africa. The questionnaires were anonymous and did not refer to the place of employment. No personal information was collected. The study sample consisted of 587 respondents. The survey was conducted in English. Table 1 shows the demographic and professional characteristics of the first-stage participants.

**Table 1:** Characteristics of the first-stage participants.

	*n*	%
*Total teaching experience*		
< 1 year	99	16.87
1–3 years	132	22.49
3–5 years	105	17.89
5–10 years	167	28.45
> 10 years	84	14.31
*Educational level*		
Secondary	333	56.73
Higher	254	43.27
*Monthly income*		
Low	267	45.49
Medium	290	49.40
High	30	5.11
*Kindergarten attribute*		
Public	286	48.72
Private	301	51.28

The second stage enrolled KG teachers practicing in the Republic of Kazakhstan (n = 105). To conduct the study, the authors contacted the centers of preschool education; 8 of them agreed to take part and provided a room for the interview. The researchers conducted interviews after the end of the working day when all the children had gone home. In each center, 5 to 18 teachers provided their answers.

### 2.3. Tools

The authors used the Symptom Checklist-90-R (SCL-90) with 9 subscales: *somatization* (distress related to one’s bodily experiences)*, obsessive–compulsive* (for intrusive thoughts and compulsive actions)*, depression* (for low mood and decreased motivation)*, anxiety* (for anxious symptoms)*, phobic anxiety* (for persistent fears)*, hostility* (for describing the feelings of anger, irritability, and resentment)*, interpersonal sensitivity* (inferiority in relationships with others)*, paranoid ideation* (for persecutory cognitions), and *psychoticism* (for interpersonal alienation and psychotic behaviors) (Coriale et al., 2019). This multidimensional questionnaire is designed to identify both clinical and non-clinical psychopathological features (Huang et al., 2020). SCL-90 has high internal consistency (Table 2). It usually takes 12 to 20 min to complete. The participants had to complete the questionnaire three times, at intervals of a month, to exclude random factors.

**Table 2:** The Cronbach’s α coefficients for SCL-90 and its subscales (Yu et al., 2020).

Somatization	0.88
Obsessive–compulsive	0.86
Depression	0.91
Anxiety	0.88
Phobic anxiety	0.81
Hostility	0.80
Interpersonal sensitivity	0.86
Paranoid ideation	0.80
Psychoticism	0.85
SCL-90	0.98

Each question was related to the respondent’s concern about a given item in the last 7 days and rated on a 5-point Likert scale from 0 (not at all) to 4 (extremely). The authors used analysis of variance to evaluate the differences in SCL-90 answers depending on teaching experience, educational level, monthly income, and kindergarten attributes. ANOVA aims to study the influence of one or more independent variables on a dependent variable. The analysis tested the multiple null hypothesis about the equality of averaged. A necessary condition for the analysis of variance is the normal distribution of the studied feature’s values in the general population. To assess the normality of the distribution, the authors used a graphical test with the frequency distribution (histogram).Statistically significant differences were determined by the F-statistics value: if the F-statistics exceeded the critical value, then the null hypothesis about the equality of averaged was rejected.

In addition to self-reports, the authors conducted a content analysis of the interviews focused on identifying the main stressors in the workplace of KG teachers and the ways to overcome them. During the content analysis, the researchers labeled respondents’ arguments and grouped them into broader categories. Each member of the research group independently listened to the transcripts of the interview and identified code phrases (codes). Then the researchers discussed the results, grouping codes by topics related to occupational stress and well-being. The theoretical basis for this stage was the paper by Faulkner et al. (2016). Accordingly, the respondents (n = 105) were asked two open-ended questions during the interview. Q1: What stresses do you face in the workplace? Q2: What coping/problem-solving means do you use?

## 3. Results

### 3.1. Psychological problems of KG teachers associated with personal and organizational factors

**Figure 1:**
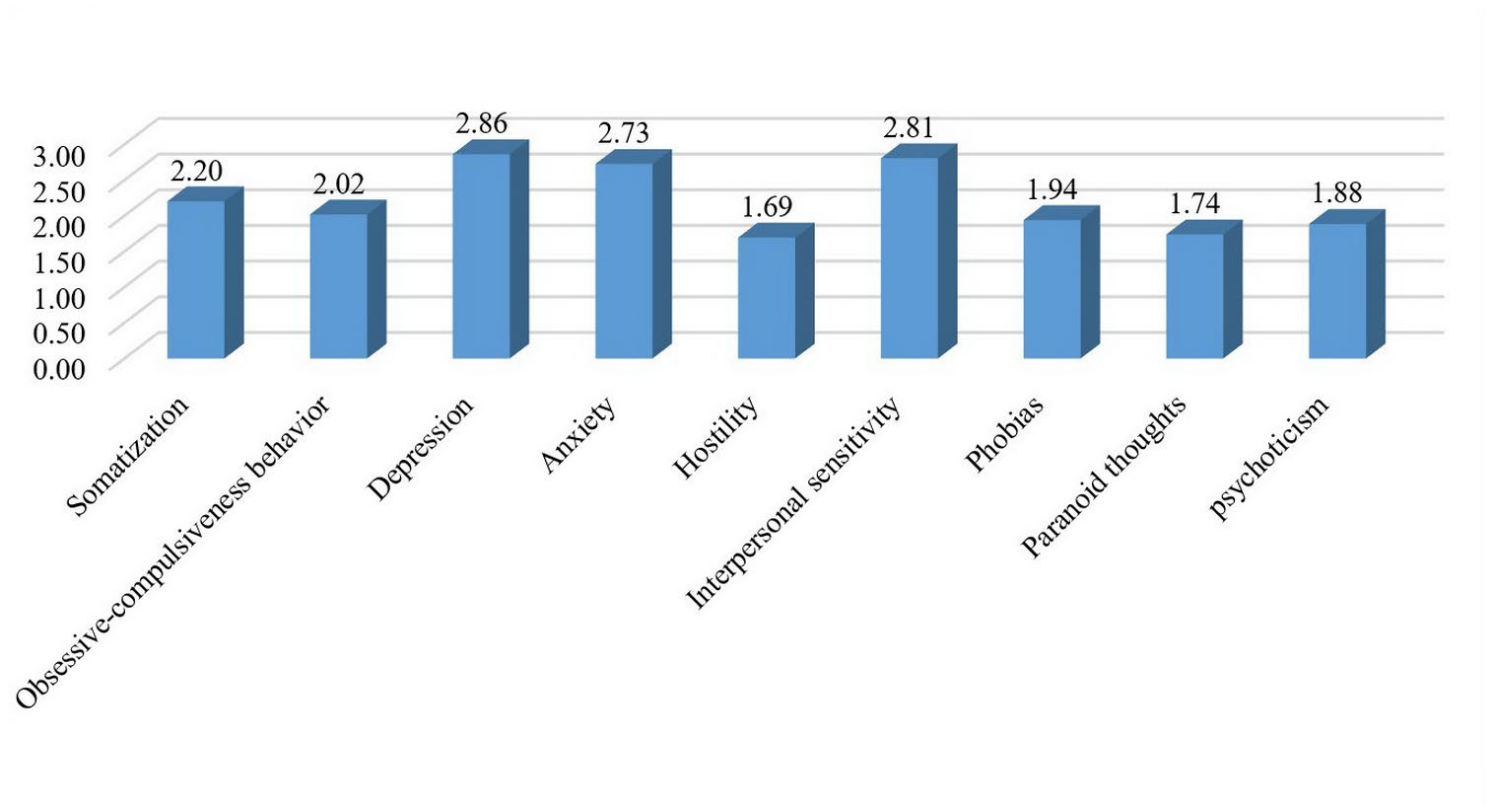
The SCL-90-C scores of the KG teachers.

The SCL-90 self-reports revealed that out of nine problems, only 4 did not significantly concern the respondents (<2): hostility, phobic anxiety, paranoid ideation, and psychoticism. Depression and interpersonal sensitivity were the leading psychological problems faced by KG teachers (Figure 1).

#### 3.1.1. Total teaching experience

KG teachers with different teaching experience had statistically significant differences in depression, anxiety, and interpersonal sensitivity (Table 3). For almost every criterion, KG teachers with experiences up to 1 year and 1–3 years had the highest scores. While depression and anxiety tended to decrease with teaching experience, interpersonal sensitivity was the lowest for 3–5 years of teaching experience but highest for the youngest and most experienced respondents. The total teaching experience was a statistically significant (at *p* < 0.05) factor of KG teachers’ mental health (*F*(2.60) = 5.99, Table 3).

**Table 3:** The descriptive statistics of mental health symptom scores and ANOVA for total teaching experience.

	Total teaching experience										
	< 1 year (n = 99)		1–3 years (n = 132)		3–5 years (n = 105)		5–10 years (n = 167)		> 10 years (n = 84)		ANOVA
	M	SD	M	SD	M	SD	M	SD	M	SD	
Mental health symptoms											
Somatization	2.32	0.53	2.22	0.66	2.03	0.6	2.34	0.69	2.1	0.77	F (2.60) = 2.04
Obsessive–compulsive	1.94	0.69	2.04	0.75	2.33	0.63	1.89	0.72	1.89	0.69	F (2.60) = 2.56
Depression	3.6	0.84	3.26	0.61	2.89	0.62	2.45	0.81	2.11	0.56	F (2.60) = 10.12*
Anxiety	3.21	0.72	3.10	0.59	2.78	0.75	2.22	0.77	2.32	0.71	F (2.60) = 12.44*
Hostility	1.85	0.56	1.88	0.55	1.56	0.63	1.51	0.76	1.67	0.73	F (2.60) = 1.21
Interpersonal sensitivity	3.47	0.7	2.56	0.63	2.22	0.8	2.78	0.65	3	0.68	F(2.60) = 21.20*
Phobic anxiety	2.36	0.68	1.92	0.68	2	0.71	1.86	0.71	1.56	0.65	F (2.60) = 1.86
Paranoid ideation	1.98	0.73	1.73	0.54	1.55	0.76	1.76	0.83	1.67	0.72	F (2.60) = 1.12
Psychoticism	1.91	0.84	1.80	0.71	2.04	0.78	1.89	0.79	1.78	0.78	F (2.60) = 1.34
SCL-90-C											F (2.60) = 5.99*

* Significant difference (*p* < 0.05).

#### 3.1.2. Educational level

In general, there were no statistically significant differences between the mental health of KG teachers by their educational level (*F* (4.49) = 0.98, Table 4). The only exception was interpersonal sensitivity, which was lower for respondents with a secondary education versus higher education (Table 4).

**Table 4:** The descriptive statistics of mental health symptom scores and ANOVA for educational level.

	Educational level				
	Secondary (*n* = 333)		Higher (*n* = 254)		ANOVA
	M	SD	M	SD	
Mental health symptoms					
Somatization	2.33	0.56	2.04	0.62	F (4.49) = 0.01
Obsessive–compulsive	2.10	0.58	1.93	0.60	F (4.49) = 0.01
Depression	2.73	0.55	2.99	0.58	F (4.49) = 1.26
Anxiety	2.81	0.49	2.65	0.57	F (4.49) = 0.02
Hostility	1.59	0.55	1.79	0.55	F (4.49) = 0.02
Interpersonal sensitivity	2.54	0.54	3.08	0.61	F (4.49) = 5.15*
Phobic anxiety	1.85	0.56	2.04	0.59	F (4.49) = 2.13
Paranoid ideation	1.8	0.6	1.68	0.58	F (4.49) = 0.16
Psychoticism	1.91	0.61	1.85	0.59	F (4.49) = 0.02
SCL-90-C					F (4.49) = 0.98

* Significant difference (*p* < 0.05).

#### 3.1.3. Monthly income

In terms of monthly income, there were differences in somatization, depression, anxiety, and interpersonal sensitivity (Table 5). At the same time, differences in obsessive–compulsiveness behavior, hostility, phobic anxiety, paranoid ideation, and psychoticism were not statistically significant in educators with different levels of monthly income. Overall, monthly income was not a statistically significant factor in KG teachers’ mental health (*F* (3.40) = 2.77, Table 5).

**Table 5:** The descriptive statistics of mental health symptom scores and ANOVA for monthly income.

	Monthly income						
	Low (*n* = 267)		Medium (*n* = 290)		High (*n* = 30)		ANOVA
	M	SD	M	SD	M	SD	
Mental health symptoms							
Somatization	2.51	1.64	2.3	1.57	1.77	1.73	F (3.40) = 3.83*
Obsessive–compulsive	2.23	1.85	1.96	1.61	1.87	1.84	F (3.40) = 2.12
Depression	3.34	1.69	3.21	1.82	2.02	1.80	F (3.40) = 5.17*
Anxiety	3.09	1.68	3.16	1.64	1.96	1.69	F (3.40) = 5.61*
Hostility	1.65	1.7	1.7	1.75	1.72	1.65	F (3.40) = 0.69
Interpersonal sensitivity	3.04	1.72	3.13	1.77	2.23	1.82	F (3.40) = 4.19*
Phobic anxiety	1.78	1.65	1.95	1.82	2.08	1.64	F (3.40) = 0.99
Paranoid ideation	1.76	1.63	1.62	1.63	1.85	1.68	F (3.40) = 0.86
Psychoticism	1.81	1.69	1.98	1.69	1.83	1.66	F (3.40) = 1.45
SCL-90-C							F (3.40) = 2.77

* Significant difference (*p* < 0.05).

#### 3.1.4. Kindergarten attribute

In terms of kindergarten attribute, there were differences in psychological problems related to somatization, obsessive–compulsive, and depression. KG teachers of private kindergartens were more susceptible to all these three indicators. Nevertheless, the general difference between teachers of private and public kindergartens was not statistically significant (*F* (4.49) = 3.32, Table 6).

**Table 6:** The descriptive statistics of mental health symptom scores and ANOVA analysis for kindergarten attribute.

	Kindergarten attribute				
	Public (*n* = 286)		Private (*n* = 301)		ANOVA
	M	SD	M	SD	
Mental health symptoms					
Somatization	1.56	0.56	2.84	0.61	F (4.49) = 5.69*
Obsessive–compulsive	1.68	0.77	2.36	0.75	F (4.49) = 7.46*
Depression	2.66	0.69	3.06	0.66	F (4.49) = 4.95*
Anxiety	2.54	0.80	2.92	0.72	F (4.49) = 2.15
Hostility	1.16	0.59	2.25	0.75	F (4.49) = 1.67
Interpersonal sensitivity	2.63	0.55	2.99	0.67	F (4.49) = 2.20
Phobic anxiety	2.14	0.76	1.74	0.68	F (4.49) = 4.36
Paranoid ideation	1.75	0.80	1.73	0.71	F (4.49) = 0.83
Psychoticism	1.79	0.69	1.97	0.65	F (4.49) = 0.64
SCL-90-C					F (4.49) = 3.32

### 3.2. Main stressors for KG teachers in the workplace during professional activities

**Figure 2:**
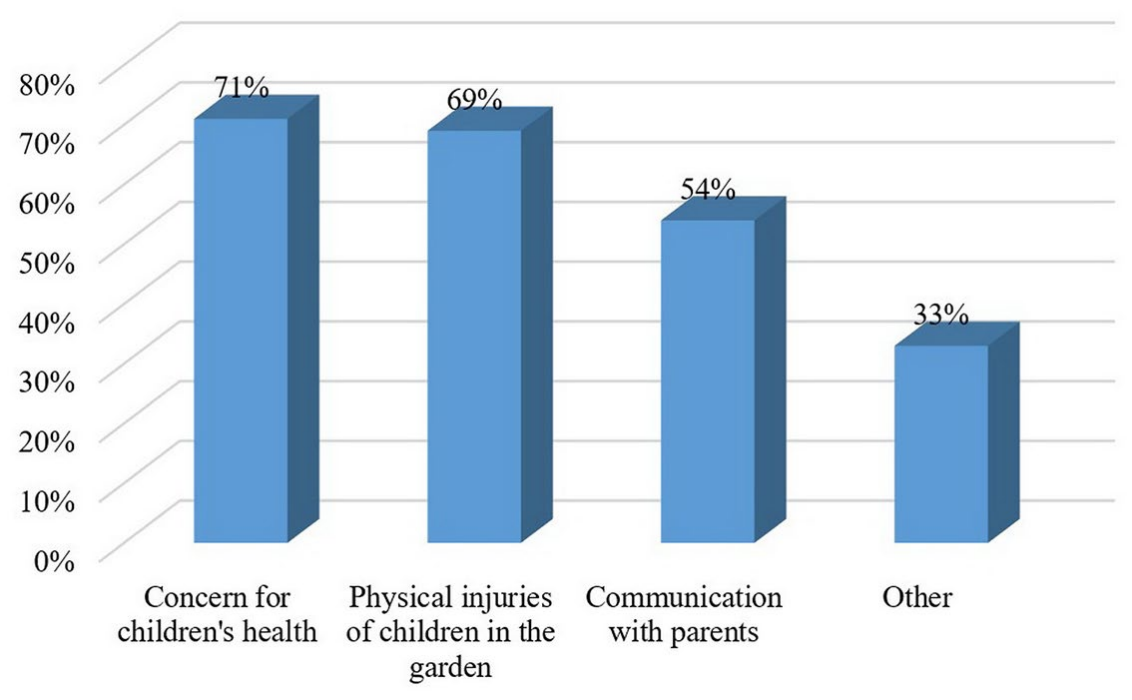
Top sources of stress in the workplace.

The main stressors were a concern for general children’s physical and mental health (71%), children’s physical injuries in kindergarten (69%), communication with parents (54%), and others (33%) (Figure 2).

#### 3.2.1. Concern for children’s health and physical injuries in kindergarten

The vast majority of respondents (71%) complained of stress due to children’s physical and mental health. They reported anxiety and tension from the fact that an unhealthy child could be brought into the group, worry about his/her condition, and that this boy or girl could infect other children. KG teachers admitted that such stress occurred almost every working day. In addition, respondents noted severe stress as a result of children’s injuries in kindergarten and emphasized that such situations were quite rare but very memorable. When one child is injured, almost all kindergarten workers feel stressed but the teacher responsible for this child is the most stressed. A possible solution may include strict control over children’s health by the kindergarten medical staff and the ability to provide first aid to an injured/sick child.

#### 3.2.2. Communication with parents

A stressor for KG teachers is the excessive anxiety of parents, which includes constant calls and messages as well as a large number of wishes and comments during personal meetings. Another stressor is the presence of disorganized parents in each team, who are constantly late, forgetting, and not continuing at home the line of education started in kindergarten with regards to personal hygiene. The respondents mentioned the passivity and disinterest of parents as a factor hindering successful interaction with them. Some respondents reported additional stress from the excessive activity of individual parents and the frequent expression of their ideas and proposals for improving the educational process and entertainment for children that do not comply with the curriculum and the internal rules of the kindergarten. However, only 22% of KG teachers expressed their desire to make efforts to improve partnerships with parents. Interaction with parents can be harmonized through training in interpersonal communication.

#### 3.2.3. Different levels of children’s independence

Children’s unwillingness to dress and use the toilet on their own and the lack of the habit of washing their hands before eating and after a walk are additional stressors for many KG teachers. At that, 42% of those who reported this predictor saw their fault in this problem and 46% had difficulty in finding mechanisms for teaching children to personal hygiene. Most likely, if a teacher is faced with such a problem, he/she has a certain gap in education, which can be filled with competently presented material in advanced training courses.

#### 3.2.4. Different levels of children’s acquirements

Only 13% of respondents reported the problem of low-performing students as the main stressor but about half of the teachers surveyed (49%) mentioned it as an additional stressor. At that, 44% of the respondents complain about the physical impossibility of an individual approach to each child. In addition, 38% of respondents were stressed due to the difficulties of raising inclusive children, who, according to doctors, can attend general groups. A possible solution may be the presence of additional qualified personnel, who can either give competent advice to the teacher or accept some responsibilities for a particular child.

#### 3.2.5. Concern for children’s safety outside kindergarten

In this research, 57% of respondents reported additional stress as a result of worrying about children from disadvantaged backgrounds when they were observed to be untidy, pouncing on food, and talking about the lack of proper nutrition at home. Also, 33% of KG teachers periodically experienced stress from the parents’ bad habits they witnessed. In addition, 16% complained that some parents sometimes came for their children being drunk.

## 4. Discussion

Thus, in this study, *concern for children’s health* and *communication with parents* were the main occupational stresses. At the same time, *different levels of children’s independence, different levels of children’s acquirements,* and *Concern for children’s safety outside kindergarten* were additional stressors for KG teachers. Faulkner et al. (2016) mentioned interaction with parents and underestimation of this profession in society as the main stressors for KG teachers associated with professional activities. Concern for children’s health was not noted as a major stressor but teachers reported that they find it difficult to care for children of all ages and worry when a child gets sick (Faulkner et al., 2016).

Such symptoms of psychological problems as depression, anxiety, and interpersonal sensitivity were ranked highest in this study. Fináncz et al. (2020) also studied the occupational and mental health of KG teachers and reported depression among ¾ of the respondents with more experienced teachers having higher depression. There was also a tendency to increase depression in the absence of social evaluation and reward (Fináncz et al., 2020). According to the current findings, more experienced KG teachers were less prone to depression but the relationship between monthly income and depression was confirmed. Educators are undoubtedly more likely to show depressive symptoms when they are in a vulnerable financial situation. Financial issues themselves are an important source of stress for people, regardless of their profession. Thus, Sinclair and Cheung (2016) called money the most important resource received from work and the most important source of stress for modern employees. In addition, monthly income refers to social factors and is a certain assessment of professional activity. Depressive symptoms can manifest in educators due to a lack of social and financial rewards (Fináncz et al., 2020).

Koulierakis et al. (2019) revealed medium and high burnout while the higher the burnout, the lower quality of life was recorded. Burnout was more frequent among elderly respondents, people with health problems, those who have undergone a redundancy or salary reduction, and those with higher education. Lower burnout of KG teachers was reported compared to their colleagues from other types of educational institutions (De Stasio et al., 2017). In European studies, almost half of KG teachers were at risk of burnout and emotional exhaustion (Clipa and Boghean, 2015; Hozo et al., 2015). The majority perceived their work as mentally burdensome and noted the lack of time for the family and unfair evaluation of their work (Čecho et al., 2019).

Faulkner et al. (2016) reported higher stress in KG teachers with lower education due to limited opportunities to learn and expand their qualifications. In this research on most SCL-90 subscales (5 out of 9, somatization, obsessive–compulsive, anxiety, paranoid ideation, and psychoticism), KG teachers with secondary education had higher scores but the superiority was not statistically significant at *p* < 0.05. American KG teachers reported that noise, isolation from other adults, and limited privacy were their additional stressors (Faulkner et al., 2016). In the current research, the respondents working in Kazakhstan reported no such predictors although it can be assumed that noise, isolation, and lack of privacy are also characteristic of their workplace. This can be explained by regional differences in mentality and character between KG teachers.

### 4.1. Practical recommendations for mental health care in KG teachers

The proposed approach to preserving the mental health and well-being of KG teachers is based on the coordinated interaction of all participants in the educational process: the teachers themselves, the KG administration, and the children’s parents.

The first step should include the working day regulation, the even workload distribution (primarily the children number in groups) among teachers, and a transparent system of remuneration and bonuses. Both experienced and novice KG teachers need financial motivation to do better.

The second step should be the use of flexible curricula that would guide the teachers’ actions and serve as the basis for organizing classes with children. Curricula oversaturated with information that are ahead of the children’s age development should not be accepted (Gallant, 2009).

The third step is to regulate the responsibilities between the KG staff and cooperation planning. The competence of each employee should be clearly defined and the intersection areas for the duties of different staff members should be documented. It would be nice to inform parents about this, e.g., to create an electronic and/or paper poster where the duties of KG employees concerning each group would be delimited and the sectors where these duties intersect would be marked. In addition, the teacher needs to have an adviser and an opportunity to get advice from another specialist, e.g., a psychologist or a doctor to immediately deal with the situation.

The fourth step should be the creation of a corporate culture and infrastructure to effectively support employees: recreation areas, joint sports and walks (excursions) after working hours, book and film clubs, master classes, seminars, and training.

The fifth step should be courses and training programs (e.g., teaching interpersonal skills and ways to counteract the stress from problematic communication with children and their parents). Also of great importance is the development of digital competence among KG teachers so that they can use technology in the preparation and conduct of classes and for communication, consultation, and interaction (Jeong and Kim, 2017; Yurinova et al., 2022). To support the digital competence of teachers around the world, there are some initiatives: United Nations Educational, Scientific and Cultural Organization (UNESCO), the European Commission (EU), the International Society for Technology in Education (ISTE), etc. (Torres-Hernández and Gallego-Arrufat, 2022).

The final step is the establishment of partnerships with parents and the search for means of mutual coordination. One example would be a mobile application proposed by Chen and Lin (2021) for developing parent-teacher communication in a remote preschool. It is worth clarifying that the solution should be individual for each team, there cannot be a universal recipe: chat is suitable for one teacher, voice messages and calls are good for another one, and the third focuses on personal consultations.

### 4.2. Research limitations

This research has some limitations. Firstly, the authors did not verify the eligibility of respondents, who identified themselves as KG teachers on the Internet and made up the first-stage sample (*n* = 587). Secondly, this study did not consider the country of respondents who participated in the first stage. Nevertheless, the situations with wages and social guarantees for educators vary in different countries, and differences in these issues between private and public kindergartens can be very significant. Thirdly, the sample of the first stage of the study was heterogeneous in some parameters: for example, there were 30 respondents with high monthly income, 290 with medium monthly income, and 267 with low monthly income. It may lead to statistical inaccuracies. Fourthly, KG teachers, who participated in the second stage (*n* = 105), worked at educational institutions of different types and their affiliation could have influenced their answers. In addition, the authors used self-report questionnaires in the first stage and open interviews in the second stage; the second stage complements and expands the first one but does not allow direct conclusions about the causal relationship between variables.

## 5. Conclusion

In this study, the authors attempted to quantify the factors that impair the mental health of KG teachers in the workplace to reasonably offer recommendations for its preservation. According to the findings, the psychological health of KG teachers is most affected by depression, interpersonal sensitivity, and anxiety (Figure 1). According to individual subscales of self-reports on psychopathological features, the most susceptible to psychological problems are less experienced, and low-income teachers working in private kindergartens. Content analysis of interviews with KG teachers about their main stressors during professional activities revealed concern for children’s health and communication with parents among the main sources of stress. The authors have proposed six specific steps to minimize stress and maintain the psychological health of KG teachers. The findings are of practical importance for administrators and officials of preschool education. Also, the paper can be considered as a theoretical basis for finding ways to protect teachers’ health. Future research may focus on the health care of nannies, who care for young children at home, or teachers, who work with inclusive students.

## Data availability statement

The raw data supporting the conclusions of this article will be made available by the authors, without undue reservation.

## Ethics statement

The studies involving human participants were reviewed and approved by The research was approved by the local ethics committees of L.N. Gumilyov Eurasian National University. The patients/participants provided their written informed consent to participate in this study.

## Author contributions

ZO, BB, UT, AM, AU, and LK: conceptualization. ZO, BB, and AM: methodology. BB, AM, and LK: software. ZO and UT: validation and investigation. AU and LK: formal analysis. AU: resources and writing—original draft preparation. BB: data curation. UT: writing—review and editing. UT and LK: visualization. ZO: supervision. BB and AM: project administration. All authors contributed to the article and approved the submitted version.

## Conflict of interest

The authors declare that the research was conducted in the absence of any commercial or financial relationships that could be construed as a potential conflict of interest.

## Publisher’s note

All claims expressed in this article are solely those of the authors and do not necessarily represent those of their affiliated organizations, or those of the publisher, the editors and the reviewers. Any product that may be evaluated in this article, or claim that may be made by its manufacturer, is not guaranteed or endorsed by the publisher.
